# Comparative Evaluation of the Differentiation and Proliferation Potential of Dental Pulp Stem Cells on Hydroxyapatite/Beta-Tricalcium Bone Graft and Bovine Bone Graft: An In Vitro Study

**DOI:** 10.7759/cureus.62351

**Published:** 2024-06-14

**Authors:** Vangmayee Shikarkhane, Vidya Dodwad, Nishita Bhosale, Swapna A Patankar, Amod Patankar, Vivek S Nair

**Affiliations:** 1 Periodontology, Bharati Vidyapeeth Dental College & Hospital, Pune, IND; 2 Oral Pathology and Microbiology, Bharati Vidyapeeth Dental College & Hospital, Pune, IND; 3 Oral and Maxillofacial Surgery, Bharati Vidyapeeth Dental College & Hospital, Pune, IND

**Keywords:** cell viability, osteogenic potential, xenograft, alloplast, mesenchymal stem cells (mscs)

## Abstract

Background: Stem cells of mesenchymal origin have good proliferative capacity when compared to other stem cell types. Dental pulp stem cells (DPSCs) are a variety of mesenchymal cells obtained from the pulpal tissue of teeth and are abundantly available and easy to obtain. DPSCs facilitate and improve the formation of new bone using different bone graft scaffolds. This present study aims to evaluate and compare the osteogenic potential of DPSCs on alloplastic and xenogeneic bone grafts.

Materials and methods: Hydroxyapatite and beta-tricalcium bone graft and bovine bone graft were used in a triplicate manner in the laboratory. DPSCs were obtained from the pulpal tissue of extracted third molars in the laboratory. The cytotoxicity, osteogenic potential, and difference in the rate of proliferation of mesenchymal cells on the biomaterials were assessed.

Results: Darker purple staining was seen in the case of hydroxyapatite/beta-tricalcium bone graft on MTT colorimetric assay stating that there was an increase in cell viability in hydroxyapatite/beta-tricalcium bone graft as compared to the bovine bone graft. Hydroxyapatite/beta-tricalcium bone graft showed more osteogenic potential as compared to the bovine bone graft as a higher degree of red staining was seen in Alizarin staining.

Conclusion: Higher cell viability and higher osteogenic proliferation and differentiation were seen on the hydroxyapatite/beta-tricalcium bone graft compared to the bovine bone scaffold.

## Introduction

The field of stem cells is an emerging and upcoming field in research. Stem cells have the unique property of transforming into different types of cells, which include bone, cartilage, nerve, and muscle cells that have excellent regenerative properties [1]. Stem cells have the capacity for both self-renewal and differentiation into several derivatives. Mesenchymal stem cells (MSCs), adult stem cells, and tissue stem cells are the various types of stem cells derived from the oral cavity [2].

Among all the different types of stem cells, MSCs have shown better medicinal and curative properties. MSCs are abundantly found in various tissues and can be procured mainly from dental pulp, bone marrow, umbilical cords, and adipose tissue. These cells possess the property of colonizing, proliferating, and differentiating locally after transplantation leading to the formation of new tissues curing the tissue dysfunction [2,3,4]. MSCs have less tendency for tumor formation and proliferation compared to induced pluripotent stem cells (iPSCs). Among MSCs, dental pulp stem cells (DPSCs) have an increased rate of proliferation and good osteogenic potential since they contain an abundance of progenitor cells. The procedure for obtaining DPSCs is less invasive as they are obtained from the pulpal tissue of extracted teeth compared to MSCs [5,6]. DPSCs can be obtained from various other sources, like the inflamed pulp of vital teeth, teeth in accessible regions with fractured crowns, shed-off deciduous teeth, and teeth extracted due to periodontal reasons, which provides a huge amount of stem cells [7]. DPSCs show high self- resumption and multipotency for the resurrection of bone defects. This has been revealed in animal models and human patients. Thus, DPSCs are an easy-to-procure and potential source of cells for treating bone defects where regenerative therapy is possible [8].

Bone grafts are routinely used in dentistry to augment bone in cases of periodontal defects, ridge augmentation, and augmenting bone around dental implants. Autografts (bone grafts taken from the same individual) act as a gold standard for excellent regeneration of bone. However, they have some disadvantages, such as morbidity of the site from where the graft is procured, difficulty in harvesting the graft, and limited availability of tissue leading to the need for search of additional options [9]. Due to these disadvantages, other options have been introduced, which include synthetic grafts known as alloplasts and animal-derived grafts known as xenografts [10,11,12]. Among alloplasts, substitutes made from biphasic calcium phosphate (BCP), and among xenografts, deproteinized bovine bone minerals (DBBMs) are commonly used due to their excellent bone regenerative potential [13].

Bone grafts containing biphasic calcium phosphate are extensively used due to their favorable properties of resorption and degradation [14]. However, some studies have shown that synthetic materials are less effective in cases where extensive and complex regeneration is required [15]. Xenogeneic bone substitutes have proved to be biocompatible and osteoconductive and are increasingly used in regenerative dentistry. These bone substitutes have certain disadvantages like transmission of infections and hypersensitivity reactions. They are obtained by removing the organic components by various chemical and physical treatments and the main component of the matrix is hydroxyapatite [16,17,18]. Some bone graft substitutes deliver viable cells for treating bone defects.

Bone graft properties like porosity, pore size, and pore interconnectivity play an important role in the cell transfer properties [11]. The success of the graft also depends on these properties. The role of stem cell interactions on bone scaffolds cannot be neglected [19,20]. For determining an ideal bone graft, the proliferation and differentiation of the stem cells in response to the scaffold need to be studied. Very few studies have been carried out in vitro to study the nature of DPSCs on bone grafts. Therefore, the aim of this study is to evaluate and compare the differentiation and proliferation potential of DPSCs on a hydroxyapatite/beta-tricalcium bone graft and bovine bone graft.

## Materials and methods

Two commercially available bone grafts: SYBOGRAF-Plus Sterile Synthetic Hydroxyapatite and beta-tricalcium phosphate bone graft composite nanocrystalline granules with a particle size of 600-700 microns and A-Oss osteoconductive bovine bone substitute with granules of 1-2 mm were used as scaffolds in this study. DPSCs of mesenchymal origin were procured from the pulpal tissue of extracted third molars. Approval for the study was obtained from the Institutional Ethics Committee of Bharati Vidyapeeth Dental College & Hospital Institutional Ethics Committee, Pune, India (approval number: EC/NEW/INST/2021/MH/0029).

Isolation of DPSCs

DPSCs used in this study were isolated using an established protocol [11]. Pulpal tissue was obtained from extracted third molars of healthy individuals in the age range of 20-30 years. According to institutional ethics guidelines, informed consent was acquired. The participants were systemically healthy, not prescribed any medications, and did not smoke or consume alcohol. After cleaning the tooth surfaces, sterile burs were used to open the pulp chamber in the cementoenamel junction region. Using barbed broaches, the tissue from the pulp region was carefully removed from the crown and root portion. One ml of collagenase type I solutions and 1 ml dispase solutions were mixed into 2 ml sterile phosphate-buffered saline (PBS) containing 100 mg/ml penicillin and 100 mg/ml streptomycin to form an enzymatic mixture in solution form.

**Figure 1:**
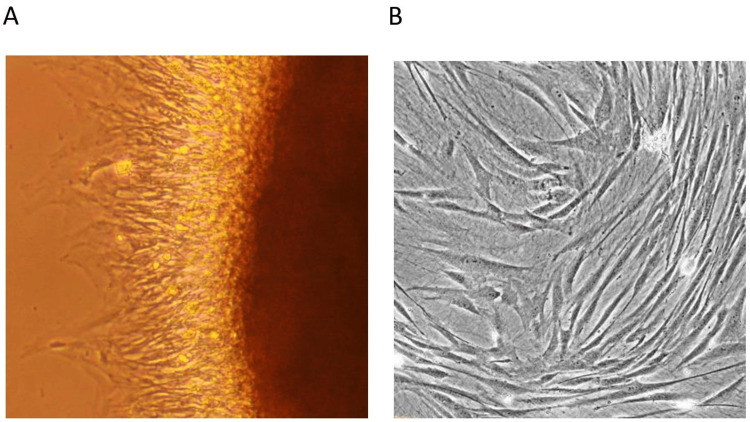
Isolation of dental pulp stem cells using an explant culture Figure A shows the explant culture under a microscope, which was obtained after mixing diced pulp tissues with a prepared enzymatic solution and allowing complete breakdown and vortexing. Figure B shows dental pulp stem cells seen under a light microscope, which were obtained from pulpal tissue in the pulp chamber of the extracted third molars.

The diced pulp tissues were then put into the prepared enzyme solution mixture for a complete breakdown for 60 minutes at 37°C. For the disintegration of the tissues, vortexing was performed every 30 minutes. The cells were then passed through a 70 µm strainer to obtain single-cell suspensions and eliminate the large cell aggregates. To abort the digestion process, 3 ml of basic minimum essential medium containing 10% (v/v) fetal bovine serum was added, and suspensions of individual cells were centrifuged at relative centrifugal force (RCF) of 67 g for five minutes at room temperature. The soluble liquid fraction obtained after centrifugation was aspirated off, and the pellets were re-suspended in 1 ml cell medium and cultured into a 25 cm^2^ cell culture flask at 37 °C in 5% CO_2_. Figure 1 shows the explant culture and DPSCs as seen under a light microscope.

Media conditioning and trypsinization

α-modification of Eagle’s medium, supplemented with 15% FBS, 100 µm L-ascorbic acid phosphate, 2 mM L-glutamine, 100 units/ml penicillin, 100 mg/ml streptomycin and 0.25 mg/ml amphotericin B were used as a medium to implant the DPSCs at a density of 104/ cm^2^ and incubated at 37°C in 5% CO^2^.

Cells were placed at 2 × 105 cells/well in six-well plates to determine the growth of cells. A hemocytometer was used to check the number of cells every 24 hours after the cells were procured from the respective wells. Then trypsinization was carried out in which trypsin was added to the cell culture flask to de-adhere the stem cells from the surface of the flask. This helped to form a colloidal solution of stem cells, which was then seated on the indicated groups.

MTT colorimetric assay

MTT colorimetric assay was used to analyze the viability of cells as a function of the redox potential of stem cells. This process involved the seeding of trypsinized stem cells in 96 welled plates at a density of 104 cells/well in conditioned Dulbecco's Modified Eagle Medium (DMEM). These wells included the culture media along with a study group, i.e., control/hydroxyapatite and beta-tricalcium bone graft/bovine bone graft.

In this technique, 50 ml of the MTT solution was added to the wells. This caused a crystallization reaction and gave a purple color to the solution. The depth of color of the solution was in proportion to the number of viable cells present.

Flow cytometry

Flow cytometry was used to examine the physical and chemical characteristics of the cells present in the study groups [21,22]. This was carried out on the seventh and 21st day after the incubation of the study groups. The cells were washed in a PBS solution and 5 ml Runx2 antibody introduced to the culture. This culture was then incubated at 4°C for 30 mins. The incubated culture was then washed in PBS solution four times over 30 minutes. FACS Calibur analysis with two lasers and four color combinations was run, and data were collected and analyzed.

Alizarin red staining

**Figure 2:**
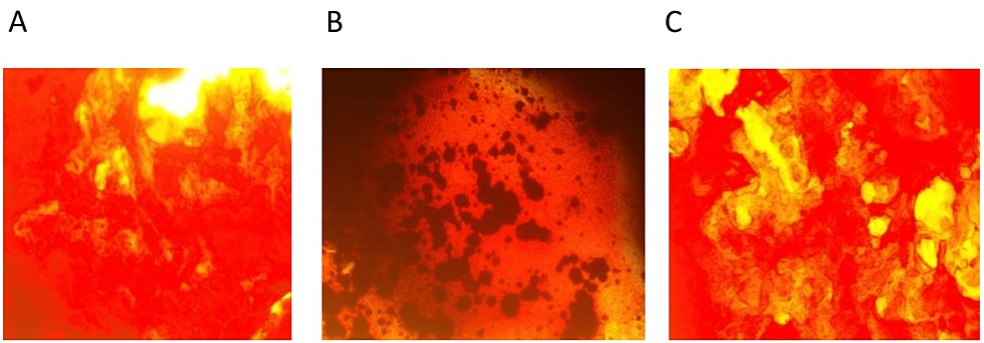
Osteogenic differentiation of dental pulp stem cells seen after alizarin red staining Figure A: Cells seen in the control group under a light microscope at 10X magnification after soaking in phosphate buffer saline and deionized water, fixing with paraformaldehyde and adding alizarin red stain. Figure B: Cells seen in the bovine bone graft group under a light microscope at 10X magnification after soaking in phosphate buffer saline and deionized water, fixing with paraformaldehyde and adding alizarin red stain. Figure C: Cells seen in the hydroxyapatite/ beta-tricalcium group under a light microscope at 10X magnification after soaking in phosphate buffer saline and deionized water, fixing with paraformaldehyde and adding alizarin red stain.

After 21 days, cells adhering to the plates were soaked in PBS and fixed in 4% paraformaldehyde for 15 minutes. The specimens were soaked twice in deionized water. One percent alizarin red 1 ml/well was added and incubated at room temperature for 20 minutes and washed four times with deionized water for 5 minutes. The cells were then viewed under a light microscope at 10X magnification. Figure 2 shows the cells as seen after alizarin red staining under a light microscope.

Statistical analysis

IBM SPSS Statistics for Windows, version 25.0 (released 2017, IBM Corp., Armonk, NY) was used for analyzing the data. Detailed statistics were expressed as means and standard deviation and numbers and percentages. Comparison between the three groups was done using one-way ANOVA followed by post-hoc Bonferroni test for pairwise comparison. In the above test, a p-value less than or equal to 0.05 was considered to be statistically significant.

## Results

**Figure 3:**
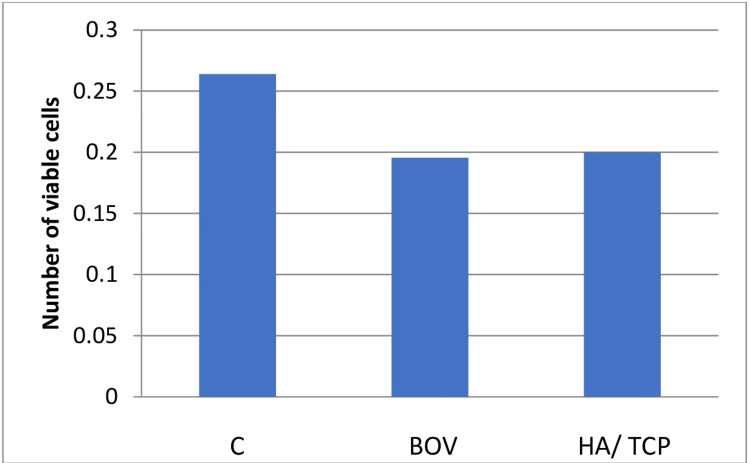
Values for cell viability for the control and test groups This graph shows the number of viable cells after assessing with an MTT colorimetric assay in the control and test groups determining the cytotoxicity of the materials.

The viability of cells was assessed using an MTT colorimetric assay. The highest cell viability was seen in the control group (0.26 ± 0.04). Among the test groups, a higher number of viable cells were seen on the hydroxyapatite/beta-tricalcium bone graft (0.20 ± 0.01) than on the bovine bone graft (0.19 ± 0.02). Figure 3 denotes the comparison between the number of viable cells in the control, BOV, and HA/ TCP groups.

**Figure 4:**
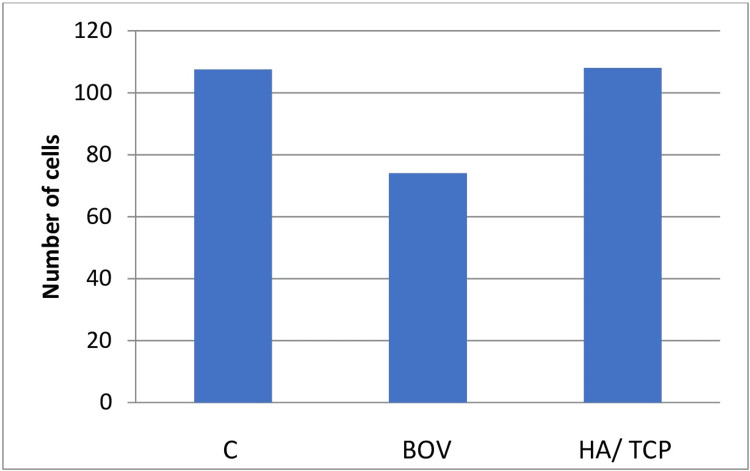
Values for cells showing osteogenic differentiation for the control and test groups This graph shows the osteogenic proliferation and differentiation potential of the cells in control and test groups, which can be determined after performing the alizarin red staining test.

Osteogenic proliferation and differentiation of the cells were higher in the hydroxyapatite/beta-tricalcium bone graft (107.99 ± 31.36) when compared to the bovine bone graft (74.02 ± 31.95). The values for the control group were similar to the hydroxyapatite/beta-tricalcium bone graft group (107.57 ± 33.37). Figure 4 denotes the comparison between the number of cells showing osteogenic differentiation in the control, BOV, and HA/ TCP groups.

## Discussion

Stem cells have always been an interesting field of research. MSCs, which can be used clinically, should be available in abundance, easily accessible, minimal invasive procedure for isolation and must have a high content of stem cells. Dental pulp tissue fulfills these criteria and thus is a preferred source to obtain stem cells for regeneration [23]. The process of isolation is simple and cost-effective and avoids tedious chemical processes.

Gronthos et al. (2000) [24] were among the first pioneers to state the term "dental pulp stem cells" (DPSCs) and successfully demonstrated that the properties of DPSCs were similar to those of mesenchymal cells. DPSCs are members of dental mesenchymal stem cells (DMSCs), possessing high multi-lineage differentiation potential, and offer an exogenous alternative to osteoblasts and other slow or non-regenerating cells. Namjoynik et al. (2023) [25] in a systematic review and meta-analysis first demonstrated that human DPSCs when combined with a scaffold showed significant bone regeneration as compared to a scaffold not seeded with cells. It is independent of the scaffold type and animal species used. DPSCs have shown promising results for treating various bone diseases, and more clinical trials need to be conducted to evaluate the effectiveness of DPSC-based therapies.

Particle size is an important factor for assessing stem cell proliferation. A particle size of 600-700 microns was used for the hydroxyapatite/beta-tricalcium bone graft group, and a similar particle size was taken for the bovine bone graft. A higher cell viability (0.20 ± 0.01) was seen with the hydroxyapatite/beta-tricalcium bone graft group when compared to the bovine bone graft group (0.19 ± 0.02). Handschel et al. (2012) [26] demonstrated in their in vitro experiments that alloplastic bone graft substitutes have specific surface properties that facilitate a flat attachment and long stretching of the cells.

In our study, we found out that higher osteogenic proliferation was seen with the hydroxyapatite/beta-tricalcium bone graft group than with the bovine bone graft group. Motamedian et al. (2017) [11] conducted a study in which proliferation, differentiation, and attachment of DPSCs to β-tricalcium phosphate (β-TCP), freeze-dried bone allograft (FDBA), and deproteinized bovine bone mineral (DBBM) were compared. The results depicted the highest potential for DPSC attachment and proliferation with β-TCP, which were similar to the results of our study. In another study, Jensen et al. (2006) [27] grafted defects in mandibles of mini-pigs with autograft, xenograft, and beta-tricalcium phosphate. On harvesting bone sections after one, two, four, and eight weeks, a higher bone formation was seen after four weeks in the autograft and beta-tricalcium groups, consistent with the results of this study.

Limitation of the study

This study was conducted in vitro on two different bone graft materials. This study alone could not prove if the same results would be seen in vivo.

## Conclusions

Our study critically evaluated and compared the differentiation and proliferation potential of DPSCs on a hydroxyapatite/beta-tricalcium bone graft and bovine bone graft. The cell viability assays showed that both bone grafts are not cytotoxic in nature and promote cell proliferation and differentiation. Both these materials cause no harm to the oral tissues. The cells showed higher osteogenic differentiation on the hydroxyapatite/beta-tricalcium bone graft, making it a better option for bone regeneration. Comparative studies with other bone grafts are necessary to arrive at any conclusive decision about the ideal bone graft. In vivo studies are also needed to analyze the properties of these materials in detail.
